# Nurses’ evaluation of critical care pain observation tool (CPOT) implementation for mechanically ventilated intensive care patients

**DOI:** 10.1016/j.dib.2019.103997

**Published:** 2019-06-14

**Authors:** Hadi Maatouk, Ahmad Al Tassi, Mirna A. Fawaz, Mohammad S. Itani

**Affiliations:** a Emergency Department, American University of Beirut Medical Center, Beirut, Lebanon; b Beirut Arab University, Faculty of Health Sciences, Beirut, Lebanon

**Keywords:** Pain, CPOT, Intensive care patients

## Abstract

Despite the fact that self-report of pain is considered the most consistent indicator of its presence, pain assessment for the critically ill mechanically ventilated patients is quite challenging, as the altered level of consciousness, sedation and the presence of life support devices commonly affect the self-report mechanism. However, in Lebanon, nearly no research articles or local professional organizations have raised this topic. Therefore, addressing and introducing the “Critical Care Pain Observation Tool” (CPOT) is of great importance and would help the healthcare providers especially “Critical Care Nurses” (CCN) in identifying and managing the patient’s hidden pain Curry Narayan, 2010. The data followed a non-experimental post-test only design to gather data from a sample of 30 critical care registered nurses where well-established psychometric instruments were used in primary data collection method, which is Critical Care Pain Observation Tool and the Feasibility and clinical utility CPOT Questionnaire. The data in this article provides demographic data about critical care nurses and their evaluation of the Critical Care Pain Observation Tool (CPOT) implementation for mechanically ventilated intensive care patients. The analyzed data is provided in the tables included in this article.

**Table:** Specifications table

Subject area	Critical Care Nursing
More specific subject area	Pain Management
Type of data	Tables
How data was acquired	Quantitative Questionnaires
Data format	Analyzed
Experimental factors	- Sample consisted of 30 critical care nurses. - Educational sessions were given to the participating nurses about the importance of pain assessment for mechanically ventilated patients, in which CPOT was introduced as the sole tool for pain assessment for these patients to be used by the staff, and they were fully instructed about how to use it. - Nurses started to use the CPOT for routine pain assessment as per hospital pain policy. In addition to that, nurses were instructed to get CPOT scores during 2 procedures that were proved painful by many researchers, which are suctioning and positioning at the beginning and at the end of the procedure.
Experimental features	The researchers measured the nurses' evaluation of the CPOT after a month of starting its implementation by using the CPOT Evaluation Questionnaire.
Data source location	Lebanon
Data accessibility	Data is available within this article
Related research article	Gélinas, C., Arbour, C., Michaud, C., Vaillant, F., & Desjardins, S. (2011). Implementation of the critical-care pain observation tool on pain assessment/management nursing practices in an intensive care unit with nonverbal critically ill adults: a before and after study. International journal of nursing studies, 48(12), 1495–1504.

**Table:** 

**Value of the data** • The data provided in this paper may be used to increase awareness about this overlooked topic. • The data shows the Critical Care Pain Observation Tool (CPOT) is an efficient instrument to detect nonverbalized pain among mechanically ventilated patients. • Further studies on CPOT would be essential to examine its effectiveness in various other hospitals and areas to generalize its adoption in practice. • Our data are concurrent with previous research studies, thus making it of interest to other researchers.

## 1 Data

Table 1 represents the demographic characteristics of critical care nurses who participated, while Table 2 shows the pain assessment profile of the nurses where it depicts that most critical care nurses do not asses pain on regular basis. Shockingly, none of the participants (n = 30) had heard about the presence of CPOT, yet 90.0%(n = 27) of the nurses had received general education regarding pain assessment and management. In addition to that, data showed that 66.7% (n = 20) rely on vital signs for pain detection. In order to assess the inter-reliability of the CCNs we first conducted the weighted kappa analysis and the intra-class correlation coefficient studies for a confidence interval of 95%. Kappa values range from −1 to +1. The higher the value of kappa, the stronger the agreement. Intra-class correlation values ranges from 0 to 1, in which the higher value closer to 1 yields the most perfect correlation and reliability of values among raters. Table 3 shows the weighted kappa analysis during position assessment. The degree to which two raters or observers, operating independently, assign the same ratings or values for an attribute being measured or observed. Three raters assessed the patients: R1, R2, and R3 (super-user). The weighted kappa score for the total CPOT score at the beginning of the positioning procedure ranged between 0.417 and 0.468 thus expressing moderate agreement score among the three different rater, having the greatest agreement between the R2 and the super-user (κ = 0.468) and the weakest agreement between R1 and the super-user (κ = 0.417). On the other hand, at the end of the procedure, the weighted kappa score for the total CPOT score ranged between 0.475 and 0.628 indicating also a moderate agreement between raters, in which the modest agreement was between R1 and the super-user (κ = 0.628) and the weakest agreement was between R2 and the super user (κ = 0.475). In addition, Table 4 shows the data of the second assessment that was done for the suctioning procedure with 150 observations. In addition, the data for the assessment of patient at the beginning of positioning procedure showed an excellent reliability with an average intra-class coefficient (ICC)of 0.945 with a 95% confidence interval from 0.912 to 0.967 (F (49,98) = 18.213, p < 0.0001) (Refer to Table 5). While at the end of the positioning procedure, the agreement of the CPOT total score was analyzed in which the average measure ICC was 0.767 showing an excellent reliability with a 95% confidence interval from 0.627 to 0.860 (F (49,98) = 4.289, p < 0.0001) (Refer to Table 6). Moreover, the data for the assessment of patient at the beginning of suctioning showed excellent reliability with an average measure of ICC was 0.964 with a 95% confidence interval from 0.942 to 0.978 (F (49,98) = 27.649, p < 0.0001) (Refer to Table 7). On the other hand, at the end of the suctioning the agreement of the CPOT total score was analyzed with an average measure of ICC of 0.882 showing also excellent reliability among the raters with a 95% confidence interval from 0.811 to 0.929 (F (49,98) = 8.475, p < 0.0001) (Refer to Table 8). Finally, Table 10 shows the Critical Care Nurses’ evaluation after they were asked to provided their feedback after the implementation of CPOT using the “CPOT Participant Evaluation Form” (see Table 9).

**Table 1:** Demographic characteristics of the participants (N = 30).

Variables	Frequency	Percentage (%)
**Age**		
Less than 25	11	36.7
26–30	12	40.0
31–35	5	16.7
More than 35	2	6.7
**Gender**		
Male	15	50.0
Female	15	50.0
**Educational level**		
Bachelor degree	26	86.7
Master’s degree	4	13.3
**Years of experience in critical care unit**		
Less than 1 year	5	16.7
2–5 years	9	30.0
More than 5 and less than 10 years	12	40.0
More than 10 years	4	13.3

**Table 2:** Pain assessment awareness among the sample (N = 30).

Criteria	Frequency	Percentage (%)
**Assess pain regularly**		
Yes	0	0
No	30	100
**Used CPOT before**		
Yes	0	0
No	30	100.0
**Received education regarding pain assessment and management**		
Yes	27	90.0
No	3	10.0
**Best way to tell whether your patient in pain**		
Vital signs	20	66.7
Behavior	9	30.0
Ventilator compliance	1	3.3

**Table 3:** Inter rater reliability for the overall CPOT score at the beginning and at the end of positioning procedure (N = 150, CI = 95%, p < 0.0001).

Weighted Kappa		
Observation	At beginning	At the end
R1 & R2	0.439 (Moderate)	0.485 (Moderate)
R1 & Super-user	0.417 (Moderate)	0.628 (Substantial)
R2 & Super-user	0.468 (Moderate)	0.475 (Moderate)

**Table 4:** Inter rater reliability for the overall CPOT score at the beginning and at the end of suctioning (N = 150, CI = 95%, p < 0.0001).

Weighted Kappa		
Observation	At beginning	At the end
R1 & R2	0.463 (Moderate)	0.477 (Moderate)
R1 & Super-user	0.582 (Moderate)	0.467 (Moderate)
R2 & Super-user	0.663 (Substantial)	1.687 (Substantial)

**Table 5:** Intra-class correlation coefficient table with 95% confidence interval with p < 0.0001 for the total CPOT score at the beginning of positioning.

Intra-class Correlation Coefficient							
	Intra-class Correlation^b^	95% Confidence Interval		F Test with True Value 0			
		Lower Bound	Upper Bound	Value	df1	df2	Sig
Single Measures	.852^a^	.776	.907	18.213	49	98	.000
Average Measures	.945^c^	.912	.967	18.213	49	98	.000

Two-way mixed effects model where people effects are random and measures effects are fixed.

a The estimator is the same, whether the interaction effect is present or not.

b Type C intra-class correlation coefficients using a consistency definition. The between-measure variance is excluded from the denominator variance.

c This estimate is computed assuming the interaction effect is absent because it is not estimable otherwise.

**Table 6:** Intra-class correlation coefficient table with 95% confidence interval with p < 0.001 for the total CPOT score at the end of positioning.

Intra-class Correlation Coefficient							
	Intra-class Correlation^b^	95% Confidence Interval		F Test with True Value 0			
		Lower Bound	Upper Bound	Value	df1	df2	Sig
Single Measures	.523^a^	.359	.672	4.289	49	98	.000
Average Measures	.767^c^	.627	.860	4.289	49	98	.000

Two-way mixed effects model where people effects are random and measures effects are fixed.

a The estimator is the same, whether the interaction effect is present or not.

b Type C intra-class correlation coefficients using a consistency definition. The between-measure variance is excluded from the denominator variance.

c This estimate is computed assuming the interaction effect is absent because it is not estimable otherwise.

**Table 7:** Intra-class correlation coefficient table with 95% confidence interval with p < 0.001 for the total CPOT score at the beginning of suctioning.

Intra-class Correlation Coefficient							
	Intra-class Correlation^b^	95% Confidence Interval		F Test with True Value 0			
		Lower Bound	Upper Bound	Value	df1	df2	Sig
Single Measures	.899^a^	.844	.938	27.649	49	98	.000
Average Measures	.964^c^	.942	.978	27.649	49	98	.000

Two-way mixed effects model where people effects are random and measures effects are fixed.

a The estimator is the same, whether the interaction effect is present or not.

b Type C intra-class correlation coefficients using a consistency definition. The between-measure variance is excluded from the denominator variance.

c This estimate is computed assuming the interaction effect is absent, because it is not estimable otherwise.

**Table 8:** Intra-class correlation coefficient table with 95% confidence interval with p < 0.001 for the total CPOT score at the end of suctioning.

Intra-class Correlation Coefficient							
	Intra-class Correlation^b^	95% Confidence Interval		F Test with True Value 0			
		Lower Bound	Upper Bound	Value	df1	df2	Sig
Single Measures	.714^a^	.589	.814	8.475	49	98	.000
Average Measures	.882^c^	.811	.929	8.475	49	98	.000

Two-way mixed effects model where people effects are random and measures effects are fixed.

a The estimator is the same, whether the interaction effect is present or not.

b Type C intra-class correlation coefficients using a consistency definition. The between-measure variance is excluded from the denominator variance.

c This estimate is computed assuming the interaction effect is absent, because it is not estimable otherwise.

**Table 9:** Data of the questionnaire about the feasibility and clinical utility of the critical-care pain observation tool (n = 30).

Question	Not at all	A Little	Uncertain	Sufficiently	Very
	(1)	(2)	(3)	(4)	(5)
Was the length of time sufficient to train to use the CPOT accurately?	0	0	0	56.7% (n = 17)	43.3% (n = 13)
Were the directives about the use of the CPOT clear?	0	0	6.7% (n = 2)	33.3% (n = 10)	60% (n = 18)
Is the CPOT quick to use?	0	3.3% (n = 1)	0	33.3% (n = 10)	63.3% (n = 19)
Is the CPOT simple to understand?	6.7% (n = 2)	0	0	23.3% (n = 7)	70% (n = 21)
Is the CPOT easy to complete?	0	16.7% (n = 5)	0	0	83.3% (n = 25)
Would you recommend using the CPOT routinely?	0	0	6.7% (n = 2)	26.7% (n = 8)	66.7% (n = 20)
Is the CPOT helpful for nursing practice?	3.3% (n = 1)	0	6.7% (n = 2)	36.7% (n = 11)	53.3% (n = 16)
Has the CPOT positively influenced your practice in assessing the patient’s pain?	0	6.7% (n = 2)	0	30% (n = 9)	63.3% (n = 19)

**Table 10:** Results of the questionnaire about the feasibility and clinical utility of the critical-care pain observation tool (n = 30).

Question	Not at all	A Little	Uncertain	Sufficiently	Very
	(1)	(2)	(3)	(4)	(5)
Was the length of time sufficient to train to use the CPOT accurately?	0	0	0	56.7% (n = 17)	43.3% (n = 13)
Were the directives about the use of the CPOT clear?	0	0	6.7% (n = 2)	33.3% (n = 10)	60% (n = 18)
Is the CPOT quick to use?	0	3.3% (n = 1)	0	33.3% (n = 10)	63.3% (n = 19)
Is the CPOT simple to understand?	6.7% (n = 2)	0	0	23.3% (n = 7)	70% (n = 21)
Is the CPOT easy to complete?	0	16.7% (n = 5)	0	0	83.3% (n = 25)
Would you recommend using the CPOT routinely?	0	0	6.7% (n = 2)	26.7% (n = 8)	66.7% (n = 20)
Is the CPOT helpful for nursing practice?	3.3% (n = 1)	0	6.7% (n = 2)	36.7% (n = 11)	53.3% (n = 16)
Has the CPOT positively influenced your practice in assessing the patient’s pain?	0	6.7% (n = 2)	0	30% (n = 9)	63.3% (n = 19)

## 2 Experimental design, materials, and methods

### 2.1 Design

A Quasi-experimental Design was implemented to answer the following research questions (1)“Is the CPOT an efficient tool to be used by critical care nurses in assessing pain of mechanically ventilated patients?” and (2) What are the Critical Care Nurses’ views towards the introduction and implementation of CPOT?“.

### 2.2 Sample and settings

The subjects were critical care registered nurse working in adult critical care unit caring for adult mechanically ventilated patients. A sample of 30 critical care nurses that fit the inclusion criteria were recruited. Critical care nurses who failed the CPOT training module and part-time adult critical care nurses were not included. The data collection extended over 4 phases from August 21, 2017 till October 14, 2017.

### 2.3 Questionnaires

Well-established psychometric instruments were used in primary data collection method, which is Critical Care Pain Observation Tool and the Feasibility and clinical utility CPOT Questionnaire. The critical-care pain observation tool “CPOT” is based on four domains: Patient’s facial expressions, Body movements, Compliance with a ventilator (or voice use for non-intubated patients), Muscle tension. The CPOT was chosen as it is a valid, reliable and clinically feasible tool adding up to Barr et al. (2013) and other researchers recommendation for the usage of this tool, besides the top management choice of this tool [1], [2], [3]. The CPOT was developed for assessing pain in critically ill adult patients unable to self-report pain. The CPOT is to be used when a patient is at rest to obtain a baseline value, during painful procedures, and before and after administering analgesics to assess the effectiveness of treatment [4]. In addition, CPOT includes four behavioral pain pointers: facial expression, body movements, compliance with ventilator and muscle tension. Each indicator is notched on a scale from 0 to 2, with the total score of 8 [5]. Feasibility and clinical utility are considered when it comes to studying the effect of implementing and launching a new practice. Feasibility is referred as the tool is simple to understand, easy to complete, and quick to use, whereas clinical utility is the ability to use the data of the tool in a useful or informative way within the clinical setting [6]. The evaluation form consists of eight closed-ended questions on a Likert scale response from 1 to 4 developed by conclusiveness made by the research team of [6], [7]. It includes questions for its ease of use, clear directions, helpful for nursing practice, help with pain assessment, adequate pain evaluation, and satisfaction with the tool. In addition, it includes a part in which nurse will be asked for any suggestions for improvement or modifications to the actual tool to adapt it to the local setting.

### 2.4 Statistical analysis

To address the objectives, data was collected for the purpose of evaluating the tool implementation. Statistical Package for the Social Sciences (SPSS) version 24 was used to examine and understand the distribution of data [8]. Data were expressed as percentages for discrete variables. Weighted kappa analysis was used to measure the inter-rater agreement among CCNs and Intra-class correlation analysis was also employed to assess the correlation between these raters. Kappa values range from −1 to +1, the higher the value of kappa, the stronger the agreement. In addition, a descriptive analysis for the Likert-type scale was used to perceive the nurse's response after using the CPOT.

## References

[1] Curry Narayan M. (2010). Culture's effects on pain assessment and management. Am. J. Nurs..

[2] Barr J., Fraser G.L., Puntillo K., Wesley E.E., Gélinas C., Dasta J.F., Jaeschke R. (2013). Clinical practice guidelines for the management. Crit. Care Med..

[3] Barr J., Fraser G., Puntillo K., Ely E., Gélinas C., Dasta J., Jaeschke R. (2013). Clinical practice guidelines for the management of pain, agitation, and delirium in adult patients in the intensive care unit. American Colleage of Crit. Care Med..

[4] Gélinas C. (2010). Nurses' evaluations of the feasibility and the clinical utility of the critical-care pain observation tool. Pain Manag. Nurs..

[5] Gélinas C., Fillion L., Puntillo K., Viens C., Fortier M. (2006). Validation of the critical-care pain observation tool in adult patients. Am. J. Crit. Care.

[6] Duhn L., Medves J. (2004). A systematic integrative review of infant pain assessment tools. Adv. Neonatal Care.

[7] Gélinas C., Arbour C. (2009). Behavioral and physiologic indicators during a nociceptive procedure in conscious and unconscious mechanically ventilated adults: similar or different?. J. Crit. Care Med..

[8] Corp I.B.M. (2016). IBM SPSS Statistics for Windows.

